# Who is responsible for follow-up after critical illness? GP, ICU and patient perspectives

**DOI:** 10.1186/s13054-025-05724-w

**Published:** 2025-11-14

**Authors:** Jonathan Stewart, Joshua Anderson, Richard Mayne, Judy Bradley, Nigel Hart, Nina Leggett, Danny McAuley

**Affiliations:** 1 Queen’s University Belfast, 97 Lisburn Road, Belfast, UK; 2 Northern Ireland Clinical Research Facility, Queen’s University Belfast, Belfast, UK; 3 Department of Critical Care, University of Melbourne, Western Health, Melbourne, Australia; 4 Regional Intensive Care Unit, Royal Victoria Hospital, Belfast, UK

## Abstract

**Background:**

Critical illness is associated with a range of physical, psychological, medical and social sequalae. It is unclear from existing clinical guidance who should be responsible for follow-up of these sequalae following hospital discharge.

**Aim:**

To explore the views of views of UK general practitioners (GPs), intensive care medicine (ICM) consultants, and patients on responsibility for follow-up care for critical illness survivors following hospital discharge.

**Methods:**

Dual methods study based in the UK. Data were collected from UK ICM consultants, GPs and patients using online questionnaires, interviews, and focus groups. Analysis was informed by the Consolidated Framework for Implementation Research (CFIR).

**Results:**

There was a lack of clarity within and between groups on who is responsible for follow-up. We identified various potential explanations for the lack of consensus including variable awareness of critical illness survivorship, ambiguity within clinical guidelines, lack of clarity on the boundaries of critical illness morbidity, evolving roles of healthcare providers, and significant workload and resource pressures within the UK healthcare system.

**Conclusion:**

The experiences of healthcare professionals and patients indicate the current lack of clarity could negatively impacting patient care and outcomes. Consensus is required on how we should define the boundaries of critical illness sequalae, and which clinical groups are responsible for care across the various transitions of care experienced by intensive care unit (ICU) survivors.

**Supplementary Information:**

The online version contains supplementary material available at 10.1186/s13054-025-05724-w.

## Background

Critical illness is usually defined as sickness which is severe enough to require advanced organ support within an intensive care unit (ICU). Between 75 and 90% of people admitted to ICU with a critical illness survive to hospital discharge [1–4]. Survivors of critical illness commonly experience long term physical, psychological, and cognitive sequalae [5], commonly known as post-intensive care syndrome (PICS), which may persist for more than five years after leaving hospital [6, 7]*.* They commonly develop new and worsening of existing medical conditions [8]. Polypharmacy is common following ICU, and is an independent predictor of poor outcomes [9]. Critical illness is also associated with significant social disruption, including financial and employment problems [10, 11].

There is currently a lack of evidence on the optimal health and social care system design to support recovery after critical illness [12–14]. In the UK, delivery of ICU follow-up services after hospital discharge varies widely, and most existing services are run by ICU staff [15]. However, general practice (GP) teams, as core primary care providers, are usually considered to be responsible for continuity and coordination of services in the community [16], and may therefore become default care providers in the absence of other support.

IN the absence of conclusive evidence, UK guidance was developed outlining potential approaches to ICU follow-up [17, 18]. This suggested responsibility for the coordination of care should sit with post ICU recovery services, led by ICU teams in the initial phase after hospital discharge, until they have discharged the patient back to the GP team. National Institute for Health and Care Excellence (NICE) guidance advises that the ICU team should “liaise with primary/community care for the functional reassessment at two to three months after the patient’s discharge from critical care”, which should be performed by an “appropriately-skilled healthcare professional(s) who is familiar with the patient’s critical care problems and rehabilitation care pathway”. Views of clinicians and patients may differ on who they think is best placed to provide care following hospital discharge. Previous research from one of the authors found neither Australian GPs nor ICU consultants felt best placed to run post-ICU follow-up services [19].

In this study, we aimed to investigate the views of UK general practitioners (GPs), intensive care medicine (ICM) consultants and patients, on responsibility for follow-up care for critical illness survivors following hospital discharge. We particularly wanted to examine previously unexplored areas including follow-up of new and existing conditions, medicine management and socioeconomic sequalae.

## Methods

### Study design

Dual methods study comprising an online questionnaire, semi-structured interviews, and focus groups. The study was informed by the Consolidated Framework for Implementation Research (CFIR) [20]. Study participants were UK GPs, ICM consultants and people with personal experience of critical illness recovery (defined as adults ≥18 years) who had experienced a critical illness (defined as requiring advanced organ support within an intensive care unit) and survived to hospital discharge.

### Data collection

#### Online questionnaire

An online questionnaire was developed to investigate GPs and ICU consultants’ perspectives (as leaders of the respective clinical teams) on who is responsible for the follow-up of critical illness sequalae following hospital discharge. The online questionnaire consisted of a clinical vignette followed by 11 questions (Supplementary Figure 1). Questions related to a range of potential sequalae of critical illness, and answers consisted of potential health and social care teams that may play a role in early and long-term patient follow-up. The questionnaire was developed and pilot tested with input and feedback from clinical and academic partners from Belfast Health and Social Care Trust and Queen’s University Belfast and was revised prior to distribution.

A link to the questionnaire was distributed between November 2022 and January 2023 to a random sample of 5000 UK GPs via Wilmington Healthcare and to a closed social media group of GPs in Northern Ireland (~1000 members). The questionnaire was disseminated by email to UK ICM consultants by the Faculty of Intensive Care Medicine (~ 2500 consultant members) and Intensive Care Society (~1,500 consultant members) via email, however there is significant overlap between these two membership databases. Potential participants were advised not to complete the questionnaire if they had already done so. We utilised convenience sampling for the questionnaire and no formal sample size calculation was completed.

The online questionnaire component of the study was reported as per the Checklist for Reporting Of Survey Studies (CROSS) standards [21].

#### Interviews and focus groups

UK GPs and ICM consultants were recruited to semi-structured interviews via the clinical and academic network of the research team. Interviews were conducted with a purposive sample of staff based on their known expertise in supporting patient recovery after critical illness (Supplementary Figure 2). Critical illness survivors were recruited via ICUSteps, a UK based charity who support patients following ICU admission [22].

For interviews we aimed to recruit up to a maximum of 20 healthcare professionals and 10 people with lived experience of critical illness. We aimed to complete two focus groups each with 5 attendees. Our sample size calculation was guided by Malterud et al, and the concept of ‘information power’ [23]. The sample size was felt to be appropriate as the interviews and focus groups had clear aims and applied an established theoretical framework to data collection and analysis.

Following consent, semi-structured interviews were conducted virtually between April and June 2023 with GPs, ICM consultants and patients via Microsoft Teams facilitated by one researcher, trained and experienced in qualitative interviewing techniques (JS). Participants were aware of JS roles as a General Practitioner and research fellow interested in improving outcomes following critical illness. Following initial analysis, two focus groups were conducted with patients in November 2023. The aim of the focus groups was to discuss the findings of the interviews, including the optimal healthcare system design to support critical illness recovery. Interviews and focus groups were recorded and transcribed verbatim into Microsoft Word.

### Data Analysis

#### Online questionnaire

Data was collected via Microsoft Forms and subsequently transferred to Microsoft Excel for analysis. Data was then transferred to R software for cleaning and analysis. The descriptive statistics of respondents are presented based on their role, region of practice and experience. Bar plots were created to summarise responses of both groups to enable comparison.

#### Interviews and focus groups

**Fig. 1:**
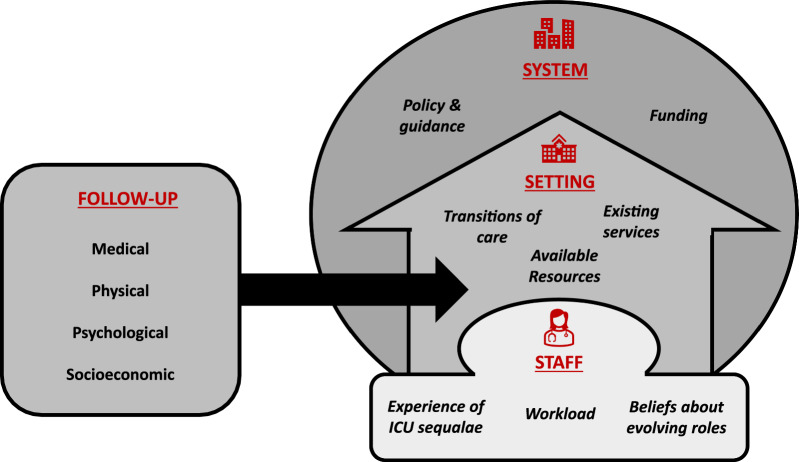
Factors influencing GP, ICU and patient perspectives on who is responsible for follow-up after critical illness

Analysis of qualitative data was conducted using framework analysis [30], which provides a five-step approach to organizing and analysing qualitative data. Data from a sample of interview and focus group transcripts were initially independently coded by two researchers (JS and RM). JS and RM are General Practitioners with experience of qualitative research methods. The domains of the CFIR [31] and Template for Intervention Description and Replication (TIDieR) provided the basis for initial framework, which was adapted and expanded (Fig. 1). Coding was compared and any disagreements discussed and resolved before the final analytical framework was applied to the full dataset.

Recruitment and data collection were ceased when data saturation was achieved, as determined using the concept of information power, and occurred when the analysis team determined no new themes were elicited from preliminary analysis of transcripts. Qualitative data were managed using NVIVO Version 20). The qualitative component of the study was reported according to the consolidated criteria for reporting qualitative research (COREQ) standards [24].

## Results

The questionnaire was completed by 152 respondents (69.1% were ICM consultants and 26.9% were GPs) (Supplementary Table 1). It was not possible to calculate exact response rates due to the overlap in distribution lists. Interviews were subsequently completed with 9 ICM consultants, 5 GPs and 13 patients. Following initial analysis 2 workshops were completed with 3 and 5 patients respectively.

### Participant views on responsibility for follow-up care for critical illness survivors following hospital discharge

There was variation between and within groups in terms of their understanding on who should provide follow-up for various potential critical illness sequalae following hospital discharge (Supplementary Figures 3a – 2 g).

#### New and existing medical conditions

GP and ICM consultant questionnaire respondents recognised a distinction between pre-existing and new medical conditions. For follow-up of the index medical condition responsible for the ICU admission (chronic obstructive pulmonary disease (COPD), in the case of the clinical vignette) and new conditions (acute heart failure and renal failure in the clinical vignette), GPs most commonly responded follow-up should be provided by a hospital specialist, GP, or community specialist team (e.g. community respiratory or heart failure team) (Supplementary Figures 3a, 3c and 3 d). Similarly, ICM consultants most commonly responded follow-up should be provided by a hospital specialist, GP, or community specialist team (e.g. community heart failure team). However, nearly 40% of ICM consultants believed they had a role in follow-up of the index condition (Supplementary Figure 3a).

ICM medical consultant interviewees also identified a distinction between ICU related medical issues, which may fall within their remit, and other pre-existing conditions;

“There are two ways of looking at it. One way is critical care, admission related medical illness, which is new and possibly indirectly or directly due to critical care and two is worsening of the underlying medical conditions.” ICU03.

“We almost certainly don’t manage comorbidities in the setting of an acute severe illness particularly well.” ICU04.

For these pre-existing conditions, there was greater agreement amongst questionnaire and interview participants that long-term follow-up was part of the *‘day job’* for GPs *(GP01)*;

“If they’re a patient living in the community in their own home and they don’t have a heart failure nurse then obviously that’s where GP is placed to assess this cohort.” GP05.

However, while over 70% of questionnaire respondents from both groups felt GPs are responsible for long-term follow-up (Supplementary Figures 3b), there was less consensus regarding early follow-up, with only 40% of GPs feeling it was their role, compared to 60% of ICU consultants.

Despite the above, some GP and ICM consultant interviewees believed there was a limited medical role during follow-up, compared to other multidisciplinary team members role in address other sequalae;

“I would say the number of discrete physical or medical problems is relatively small” ICU02.

“Our role in terms of actually managing medical problems…recently diagnosed, is very limited. There’s more of a role for the physio, the OT, for the social worker” GP01.

#### Medications

There was greater consensus on who is responsible for follow-up of new and existing medications following hospital discharge, with over 60% of GPs and ICM consultants viewing this as the role of GPs early following hospital discharge (Supplementary Figures 3e). However, ICM consultant interviewees recognised reconciliation of new medication is part of their role during hospitalisation, and this does not always occur;

“We’re often quite bad in ICU at stopping or telling the sort of downstream carers why we’ve sometimes started these drugs and when we think they should be stopped.” ICU05.

Patients also highlighted medicines reconciliation does not always occur;

“I’m still on such and such a drug, and the medics were saying “you should have been off that within four weeks” and this was two to three years down the line” (P06).

#### Physical weakness

There was a greater degree of uncertainty regarding which team was responsible for arranging follow-up of new functional impairments such as physical weakness (Supplementary Figure 3f). Nearly 60% of ICM consultant and GP questionnaire respondents believed community teams (e.g. physiotherapy) should provide early follow-up. However, nearly 50% of ICM consultants also believed it was within their remit to arrange early follow-up, compared to less than 15% of GPs seeing this as a role of ICU teams.

#### Socioeconomic sequalae

For follow-up of new social and financial issues, questionnaire respondents most commonly believed this should be done by social workers (around 40% of GPs and 50% of ICM medical), however nearly a third of both groups believed it fell within the remit of GPs (Supplementary Figure 3 g).

### Potential explanations for lack of clarity on remit and responsibility

We identified several potential explanations for the lack of clarity on remit and responsibility following hospital discharge seen within quantitative and qualitative data.

- 1) Understanding of evolving roles

GPs and ICM consultants acknowledged they lacked understanding of each other’s roles. Some ICM consultants perceived GPs as the default providers following hospital discharge and would signpost patients to see their GP for certain issues. This conflicted with some GPs who believed the default position should be secondary care teams arranging any required follow-up where possible;

“Just as long as they’re not sending me letters. ‘Please do an ECG in this patient’…Do it yourself.” GP01.

GPs, ICM consultants and patients highlighted that the long-term relationship between GPs and their patients could be harnessed to ensure continuity of care, care coordination, facilitation of recovery and prevention of deterioration following hospital discharge.

“You’re still responsible for coordinating care in the community.” GP03.

“Trying to prevent deterioration and getting people that sort of the appropriate care. I just don’t really see how anybody could say that’s not it’s not the role of a GP” GP05.

“So many different specialties. Different departments talking to me on their bit, but not as a whole and my GP, thank goodness, is brilliant, but had I not had a good GP that listened to me and connect all the pieces, I’d be utterly lost.” P08.

However, participants recognised that the paradigm of a single-family GP who knows each patient and their family is evolving;

“Nowadays one doesn’t have GP. There’s a team of people and you get the person who happens to be available on that day.” P06.

“They don’t always see the familiar family doctor that they may have done in generations gone by.” ICU04.

Some GPs believed the lack of clarity on the boundaries of their remit had increased with the evolution of their role;

“You will get I suppose different views… the role is changing quite a lot, and people aren’t really sure what the role of the GP is at the moment… people might be annoyed at getting away from that kind of old way.” GP02.

- 2) Awareness of critical illness sequalae

Patients and ICM consultants commonly believed that GPs had variable experience of common ICU sequalae, and this impacted the care patients received following hospital discharge;

“That sense of the GP not really knowing what to do. Not really having that experience” P12.

“How well does a GP understand the sequelae of critical illness or critical care?” ICU02.

“A lot of patients end up just back in primary care with no real coordination of all of the care that they need and with primary care providers, who I guess, have got varying degrees of experience of these sorts of patients.” ICU05.

- 3) Understanding of care delivery in the community

While ICM consultants may have greater understanding of ICU sequelae, they may also lack understanding of how patients can access the services to address these complications or the impact of the patient’s social context;

“My understanding the primary care is gonna be very limited” ICU 04.

“An awful lot of hospital-based consultants have absolutely no understanding of the environment that patients live in and how that impacts upon their ability to recover.” ICU 04.

- 4) Healthcare system capacity

Another important potential driver for GP views on whether they are responsible for provision of follow-up of survivors of critical illness is their pre-existing workload;

“We obviously don’t have time or resources to contact these people individually.” GP03.

Some GPs highlighted the importance of their funding model, and the need for additional funding and resources if GPs are expected to provide follow-up care beyond that which is covered by their existing contractual arrangements;

“If we’re going above and beyond that… We need to look at the resource. We need to look at the planning” GP04.

Other GPs highlighted that given critical illness survivors are a relatively rare high risk patient cohort, their care should be prioritised despite limited available resources;

“I think it’s it would be reasonable if it’s something you deal with day-to-day. These are our high-risk patients, so I don’t see why that would be an annoyance to add to your workload.” GP02.

GPs agreed that any follow-up they were asked to provide should add value to the patients care journey;

“If it’s meaningful work… and make a difference to this patient, I’m very happy to do it.” GP01.

There was also recognition of the limited capacity within hospital services. Some ICM consultants viewed delivery of care in primary care as more cost effective;

“so much depends on things like the resources available” ICU05

“Primary care is gonna be cheaper than secondary care” ICU07

Patients also highlighted the impact lack of resources had on ability of hospital and GP services to provide follow-care;

“A lot of it’s down to the hospital staff are overstretched. They haven’t got the time to spend with the patients. And when you’re in the community, at the GP, they haven’t got time to sit down and go through it in fine detail with you.” P09.

- 5) Transitions of care

Patients, GPs and ICM consultants all acknowledged multiples issues related to transitions of care between ICU, inpatient hospital wards and the community;;

“Transitions between care are particular fault lines….Quite often these are complex patients who’ve got multiple complex needs that don’t easily fit into one specialist domain.” ICU02.

ICM consultant interviewees highlighted the potential pitfall of moving from the generalist ICU setting to a specialist hospital ward;

“When you’re in intensive care, you get a very general approach to your care because I guess we’re all generalists, but as soon as the patient no longer needs critical care, you end up with some sort of ology looking after you” ICU 05.

GPs highlighted that their capability to deliver high quality care following transition from hospital to home was often limited by inadequate communication from secondary care, and where communication did occur there was often an unrealistic expectation that GPs would carry out follow-up that either didn’t fall within their remit or could have been arranged by secondary care.

“It’s about good communication. It’s about a consistent approach to that, and it’s about the GP feeling that they’re part of a continuum of care” GP04.

ICU consultants also recognised communication can be inadequate;

“We often signpost to the GP and then don’t give them the information that they need.” ICU 04.

Some GPs recognised their presence across the continuum of care and existing knowledge of the patients may enable them to identify patients who are particularly vulnerable following hospital discharge such as self-employed, socio-economically deprived, low health literacy or inadequate social support.

## Discussion

Research over the past three decades has identified an increasingly diverse range of potential sequalae that critical illness survivors can experience compared to other patients. The evidence for how best to mitigate these complications is lacking, particularly following hospital discharge. Existing guidelines, from both within and outside the ICU context, advocate for provision of certain follow-up [17, 18]. However, it is often ambiguous regarding which clinical group is responsible for arranging and delivery of this follow-up. This study aimed to investigate the views of UK general practitioners (GPs), intensive care medicine (ICM) consultants and patients, on responsibility for follow-up care for critical illness survivors following hospital discharge.

We found significant variation within and between speciality groups, and views often diverged significantly from the existing guidance [17, 18]. There was relative consensus that existing medical problems and medications continued to fall within the remit of General Practice and once optimised the long-term follow-up of these issues fell within the remit of GPs. However, there was considerable variation in terms of the views of who was responsible for optimising the care of new medical conditions and medications. This is important and may help explain why multimorbidity (the presence of two of more medical conditions) and polypharmacy (the prescribing of multiple medications, often 5 or more) are some of the strongest predictors of hospital readmission [8, 25–28]. Our own previous work identified a variety of patient and healthcare system factors which might explain why multimorbidity is associated with worse outcomes following critical illness [8]. Social context appears to play a particularly important role. Socioeconomic sequalae of critical illness, and the impact of psychosocial factors on critical illness outcomes, have gained significant recent interest [11, 29, 30]. Participants in this study believed social workers should take the lead on follow-up of socio-economic sequalae. However, they also believed GPs are in a unique position to understand how a patient’s psycho-social context impacts their health outcome, which corresponds with previous work on the core attributes of general practice [16].

New physical weakness was one of the most contentious sequalae. Unlike new and worsened existing medical conditions, physical weakness is now more widely recognised as a direct consequence of critical illness within what is often known as post intensive care syndrome (PICS) [31]. The discrepancy between GP and ICU teams may relate to lack of GP recognition of PICS and the varied existing services which have been developed across the UK, usually by ICU teams, to mitigate it [15, 32–35]. This also highlights the potential limitations of PICS in diverting attention from wider critical illness sequelae [17, 36]. There is currently no consensus on how broadly we define ‘critical illness sequalae’. When it was initially coined, PICS focused on physical, mental health and cognitive problems which could be directly attributed to critical illness, including treatment [37]. Previous research has demonstrated a lack of awareness amongst GPs of these complications [32, 33, 35]. As already discussed, there is increasing acceptance that critical illness has much broader determinants and sequalae including multimorbidity, polypharmacy and socio-economic factors [38], which commonly fall within the remit of general practice. However, there is a lack of evidence from clinical trials for on which general practice led interventions to address these problems and lead to improved patient outcomes [16, 39–41]. It is vital to have clarity on the boundaries of critical illness sequalae, and whether we include wider health and social care consequences.

Historically GPs have been considered responsible for continuity and coordination of care in the community [42, 43]. Previous research indicates continuity of care in general practice leads to better health outcomes, including lower mortality rates, fewer hospital admissions, improved patient experience, and more cost-effective care [44, 45]. However, participants in the current study highlighted that the well documented workload pressures in UK general practice have led to a more transactional model of General Practice in the UK, which is likely inadequate to meet the needs of this high-risk patient cohort of critical illness survivors. Previous research suggests even when GPs work alongside ICU staff to prioritise critical illness survivor this may not improve outcomes. A previous study which evaluated a primary care based intervention which combined case management by ICU nurses and support for primary care physicians did not find any improvement in mental health related outcomes among survivors of sepsis and septic shock [46]. However, there is also a lack of evidence of ICU team led follow-up, and a recent study found an intensivist-led multidisciplinary model of follow-up following hospital discharge was associated with worse quality of life one year after ICU discharge [47].

We likely need to rethink how we deliver care for this patient cohort. If we continue to make assumptions about the role of certain professional groups in regard to provision follow-up, including the ubiquitous role of general practitioners, there will continue to be significant variation in care provision for patients. In our previous work which examined factors to consider when designing the optimal approach to supporting ICU recovery following hospital discharge, we identified the need to consider how best to balance the development of new bespoke services, which may not be compatible with or cost-effective for healthcare systems, against integration with existing healthcare systems including the patients GP, which may not be readily available due to lack of resources [48]. This study provides further evidence that future intervention designs should consider how to best harness this balance across the continuum of care to ensure a personalised holistic approach which identifies and addresses unmet patient needs, and which is compatible with existing healthcare systems.

Finally, as already highlighted there is currently a lack of evidence on the optimal health and social care system design to support recovery after critical illness, and whether bespoke follow-up care after critical illness improves patient outcomes [12–14]. This should clearly be prioritized alongside implementation considerations regarding who is responsible for implementation in routine clinical care.

### Strengths and limitations

Integration of data from quantitative and qualitative sources enabled us to gain more in-depth perspectives from clinicians and patients located in different regions across the UK. Templates for semi-structured interview and focus group questions were informed by the responses to the initial questionnaire, which provided deeper qualitative insights. ICM consultants, GPs and patients who have experienced being discharged from ICU are all key stakeholders in defining how to deliver optimal care to patients being discharged into the community post ICU. However, staff who agreed to participate may have represented a group who are more experienced with sequalae of critical illness, or with more positive attitudes towards it. There was a low response rate to the online questionnaire from GPs, which impacted the generalisability of the findings. This may reflect the identified workload and funding pressures within primary care currently or could be due to a lack of interest and engagement amongst GPs in this area. Furthermore, we did not include the views of hospital and community specialist consultants (e.g. physicians or surgeons) who play an important role in leading the provision of follow-up following hospital discharge. Their role should be the focus of future work.

## Conclusions

There is a lack of clarity regarding which professional groups are responsible for arranging and delivering follow-up care for the various complex and interconnected sequalae experienced by ICU survivors. We identified various potential explanations including a lack of understanding of professional roles and responsibilities, lack of capacity and workload pressures within the UK healthcare system, and a lack of clarity on how broadly we define the boundaries of critical illness sequalae. Further work is required involving all stakeholders to obtain consensus on the boundaries of professional responsibilities and critical illness sequalae, and to design and test comprehensive pathways of care which identity and address unmet patient needs, with the aim of improving outcomes for patients and their families.

## Supplementary Information

Supplementary materials 1

Supplementary materials 2

Supplementary materials 3

## Data Availability

Raw data from this project will not be made available.
