# Supplementary material for: Who is responsible for follow-up after critical illness? GP, ICU and patient perspectives
Source: Crit Care. 2025 Nov 14;29:489. doi: 10.1186/s13054-025-05724-w (PMC12619521; doi:10.1186/s13054-025-05724-w)
Supplement: Supplementary file 1 — Supplementary materials 1 [file 13054_2025_5724_MOESM1_ESM.docx]

**Supplementary Figure 1 – Clinical vignette for questionnaire**

| **GP and ICU consultant views on their role after ICU admission**  People have complex needs after being in the Intensive Care Unit (ICU). This study aims to explore the views of General Practitioners and Intensive Care Medicine Consultants on their role after a person is discharged from hospital following an admission to the ICU.  The study is being undertaken by Queen’s University Belfast (QUB).   Any General Practitioner or Intensive Care Medicine Consultant currently practicing in the United Kingdom (UK) is eligible to take part.   Your responses to this survey will be anonymous. The information you provide will be confidential and stored in a safe manner in the Wellcome-Wolfson Centre for Experimental Medicine (QUB). Staff at the QUB Research Governance Office may review the data collected in this study for the purposes of auditing research quality. The results of this study may be published and used for educational purposes.  **Section 1**  1.What is your role? Multiple choice.   - General Practitioner (GP) - Partner - General Practitioner (GP) - Salaried - General Practitioner (GP) - Locum - Intensive Care Medicine Consultant - Intensive Care Medicine Consultant – with involvement in ICU follow-up services   2.In which region are you currently practicing? Single choice.   - England - Wales - Scotland - Northern Ireland   3.Do you have personal experience of caring for patients after they are discharged from hospital following an admission to the Intensive Care Unit (ICU)?Single choice.   - Yes, regularly (daily or weekly) - Yes, some (monthly) - Yes, rarely (annually) - No   **Section 2**  **Scenario**  A single scenario is presented. Please indicate your opinion on the subsequent questions.  *A 56-year patient was intubated and ventilated in the intensive care unit (ICU) following an acute exacerbation of their COPD. Prior to hospital admission, their past medical history included moderate COPD, hypertension, and type 2 diabetes, which were all diagnosed and under review by their GP.*   *During ICU admission, the patient developed several complications including acute kidney injury and acute heart failure. Echocardiography revealed reduced left ventricular systolic function. They were commenced on several medications during their ICU stay including Longtec. Following a 2-week period of mechanical ventilation in the ICU, and a subsequent 3-week admission to a general medical ward, the patient was discharged home. At hospital discharge, the patient’s renal function had improved since leaving the ICU, but remained deranged compared to their baseline (eGFR = 35, Baseline = >60).*   *At home the patient suffered from physical weakness and severe fatigue. They were previously self-employed and were unable to work for 3 months after hospital discharge.*  4.What follow-up, if any, is required for this patient’s COPD following hospital discharge?  Please select all that are relevant. Multiple choice.   - None - Early follow-up by the ICU team - Early follow-up by the hospital ward team - Early follow-up by a hospital specialist team (e.g. respiratory outpatient clinic) - Early follow-up by the patient's GP team - Early follow-up by a community team (e.g. community respiratory team) - Early follow-up by a social work team - Long term follow-up by the ICU team - Long term follow-up by the hospital ward team - Long term follow-up by a hospital specialist team (e.g. respiratory outpatient clinic) - Long term follow-up by the patient’s GP team - Long term follow-up by a community team (e.g. community respiratory team) - Long term follow-up by a social work team   5.What follow-up, if any, is required for this patient’s other existing medical conditions (i.e. diabetes and hypertension), following hospital discharge?  Please select all that are relevant. Multiple choice.   - None - Early follow-up by the ICU team - Early follow-up by the hospital ward team - Early follow-up by a hospital specialist team (e.g. endocrinology outpatient clinic) - Early follow-up by the patient's GP team - Early follow-up by a community team (e.g. community diabetes team) - Early follow-up by a social work team - Long term follow-up by the ICU team - Long term follow-up by the hospital ward team - Long term follow-up by a hospital specialist team (e.g. endocrinology outpatient clinic) - Long term follow-up by the patient’s GP team - Long term follow-up by community team (e.g. community diabetes team) - Long term follow-up by a social work team   6.What follow-up, if any, is required for this patient’s new renal impairment following hospital discharge?  Please select all that are relevant. Multiple choice.   - None - Early follow-up by the ICU team - Early follow-up by the hospital ward team - Early follow-up by a hospital specialist team (e.g. nephrology outpatient clinic) - Early follow-up by the patient's GP team - Early follow-up by a community team - Early follow-up by a social work team - Long term follow-up by the ICU team - Long term follow-up by the hospital ward team - Long term follow-up by a hospital specialist team (e.g. nephrology outpatient clinic) - Long term follow-up by the patient’s GP team - Long term follow-up by a community team - Long term follow-up by a social work team   7.What follow-up, if any, is required for this patient’s new heart failure following hospital discharge?  Please select all that are relevant. Multiple choice.   - None - Early follow-up by the ICU team - Early follow-up by the hospital ward team - Early follow-up by a hospital specialist team (e.g. cardiology outpatient clinic) - Early follow-up by the patient's GP team - Early follow-up by a community team (e.g. community heart failure team) - Early follow-up by a social work team - Long term follow-up by the ICU team - Long term follow-up by the hospital ward team - Long term follow-up by a hospital specialist team (e.g. cardiology outpatient clinic) - Long term follow-up by the patient’s GP team - Long term follow-up by a community team (e.g. community heart failure team) - Long term follow-up by a social work team   8.What follow-up, if any, is required for this patient’s new medications started in ICU (e.g. Longtec) following hospital discharge?  Please select all that are relevant. Multiple choice.   - None - Early follow-up by the ICU team - Early follow-up by the hospital ward team - Early follow-up by a hospital specialist team (e.g. pain outpatient clinic) - Early follow-up by the patient's GP team - Early follow-up by a community team (e.g. community pharmacy team) - Early follow-up by a social work team - Long term follow-up by the ICU team - Long term follow-up by the hospital ward team - Long term follow-up by a hospital specialist (e.g. pain outpatient clinic) - Long term follow-up by the patient’s GP team - Long term follow-up by a community team (e.g. community pharmacy team) - Long term follow-up by a social work team   9.What follow-up, if any, is required for this patient’s new physical weakness following hospital discharge?  Please select all that are relevant. Multiple choice.   - None - Early follow-up by the ICU team - Early follow-up by the hospital ward team - Early follow-up by a hospital specialist team (e.g. physiotherapy outpatient team) - Early follow-up by the patients GP team - Early follow-up by a community team (e.g. community physiotherapy team) - Early follow-up by a social work team - Long term follow-up by the ICU team - Long term follow-up by the hospital ward team - Long term follow-up by a hospital specialist team (e.g. physiotherapy outpatient team) - Long term follow-up by the patient’s GP team - Long term follow-up by a community team (e.g. community physiotherapy team) - Long term follow-up by a social work team   10.What follow-up, if any, is required for this patient’s new employment disruption following hospital discharge?  Please select all that are relevant. Multiple choice.   - None - Early follow-up by the ICU team - Early follow-up by the hospital ward team - Early follow-up by a hospital specialist team - Early follow-up by the patients GP team - Early follow-up by a community team - Early follow-up by a social work team - Long term follow-up by the ICU team - Long term follow-up by the hospital ward team - Long term follow-up by a hospital specialist team - Long term follow-up by the patient’s GP team - Long term follow-up by a community team - Long term follow-up by a social work team   11.For this patient, please list the top 3 priorities following hospital discharge. Multiple choice.   - Please select at most 3 options. - None - Follow-up of this patient’s COPD - Follow-up of this patient’s other existing medical problems (i.e. hypertension and diabetes) - Follow-up of this patient’s new renal impairment - Follow-up of this patient’s new heart failure - Follow-up of this patient’s new medications - Follow-up of this patient’s new physical weakness - Follow-up of this patient’s employment disruption |
| --- |

***Supplementary Figure 2 - Qualitative interview schedule and Focus Group Schedule***

| Q1: What is you view on the proposed approach to address new and existing medical conditions?   - Prompt: Overall approach? approach to prioritising conditions? approach to management? - Prompt: Is there anything that should be changed, added or removed? - Prompt: Are there any challenges for delivery?   Q4: What is you view on the proposed approach to address multiple medications (polypharmacy)?   - Prompt: Is there anything which should be changed, added or removed? - Prompt: Are there any challenges for delivery?   Q3: What is you view on the proposed approach to address social and economic problems?   - Prompt: Is there anything which should be changed, added or removed? - Prompt: Are there any challenges for delivery?   Q4: For the location of the recovery programme (i.e. at home, hospital outpatient department, in the community, or combination), what is you preferred option, and why?   - Prompt: What are the benefits of this option over the others? - Prompt: Are there any challenges for this option?   Q5: For how the recovery programme is delivered (i.e. virtual, face-to-face care or combination), what is you preferred option, and why?   - Prompt: What are the benefits of this option over the others? - Prompt: Are there any challenges for this option?   Q6: For the timing of delivery of the recovery programme (i.e. at hospital discharge, within first few weeks, later), what is you preferred option, or options, and why?   - Prompt: What are the benefits of this option over the others? - Prompt: Are there any challenges for this option? |
| --- |

***Supplementary Table 1 - Characteristics of questionnaire respondents***

| **Characteristics of respondents** | | **Frequency** | **%** |
| --- | --- | --- | --- |
| What is your role? | General Practitioner | 41 | 26.9 |
|  | Intensive Care Medicine (ICM) Medical Consultant | 105 | 69.1 |
|  | Other | 6 | 4.0 |
| What region are you from? | England | 110 | 72.3 |
|  | Wales | 4 | 2.6 |
|  | Scotland | 8 | 5.3 |
|  | Northern Ireland | 27 | 17.8 |
|  | Other | 3 | 2 |
| Do you have experience of supporting patients after critical illness following hospital discharge? | Yes, regularly (daily or weekly) | 7 | 4.6 |
|  | Yes, some (monthly) | 28 | 18.4 |
|  | Yes, rarely (annually) | 55 | 36.2 |
|  | No | 62 | 40.8 |

**
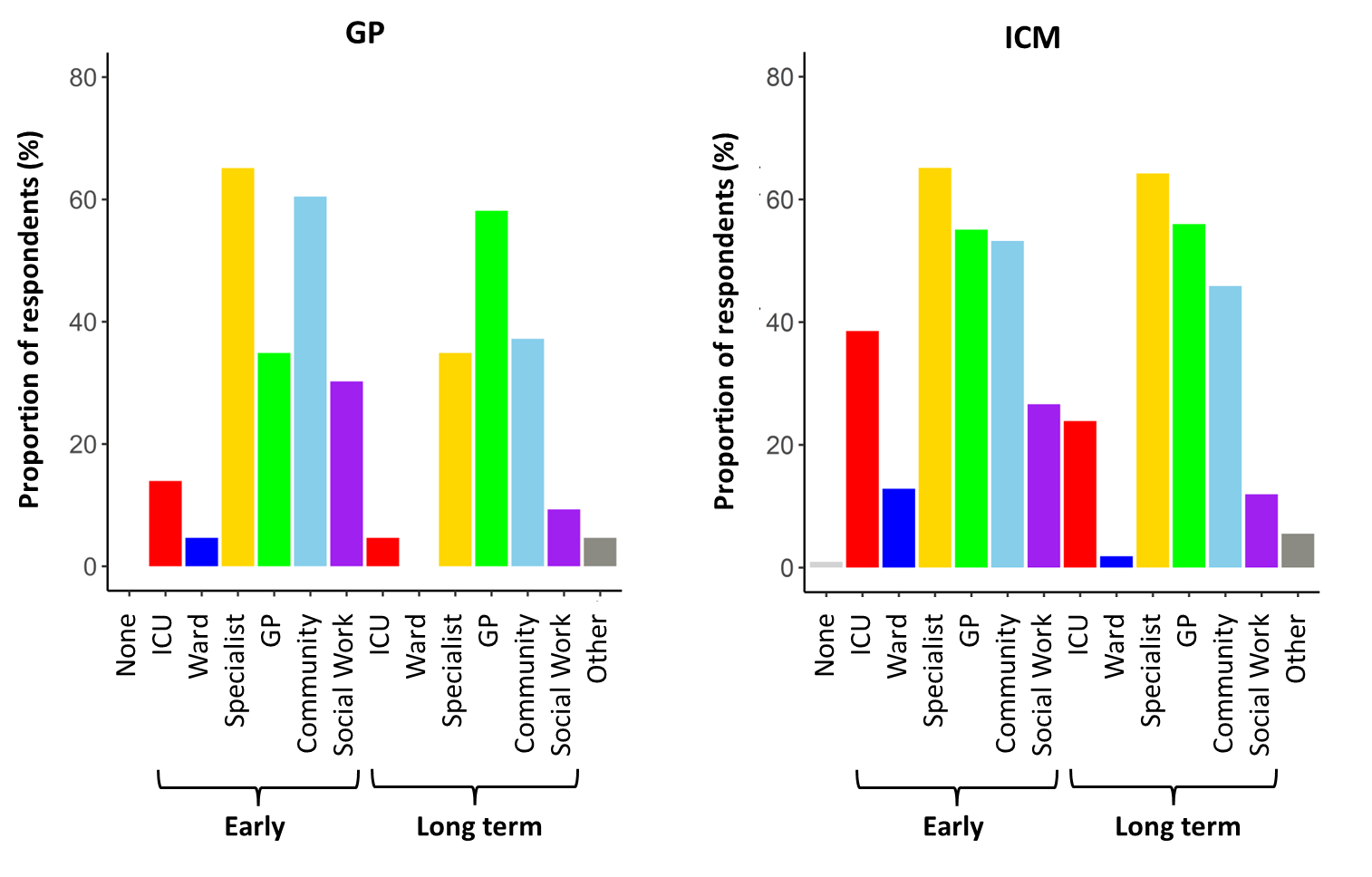
**

**Supplementary Figure 3a – Reponses to online questionnaire “Who do you think is responsible for follow-up of the condition responsible for ICU admission (in this case COPD)?”.**

**
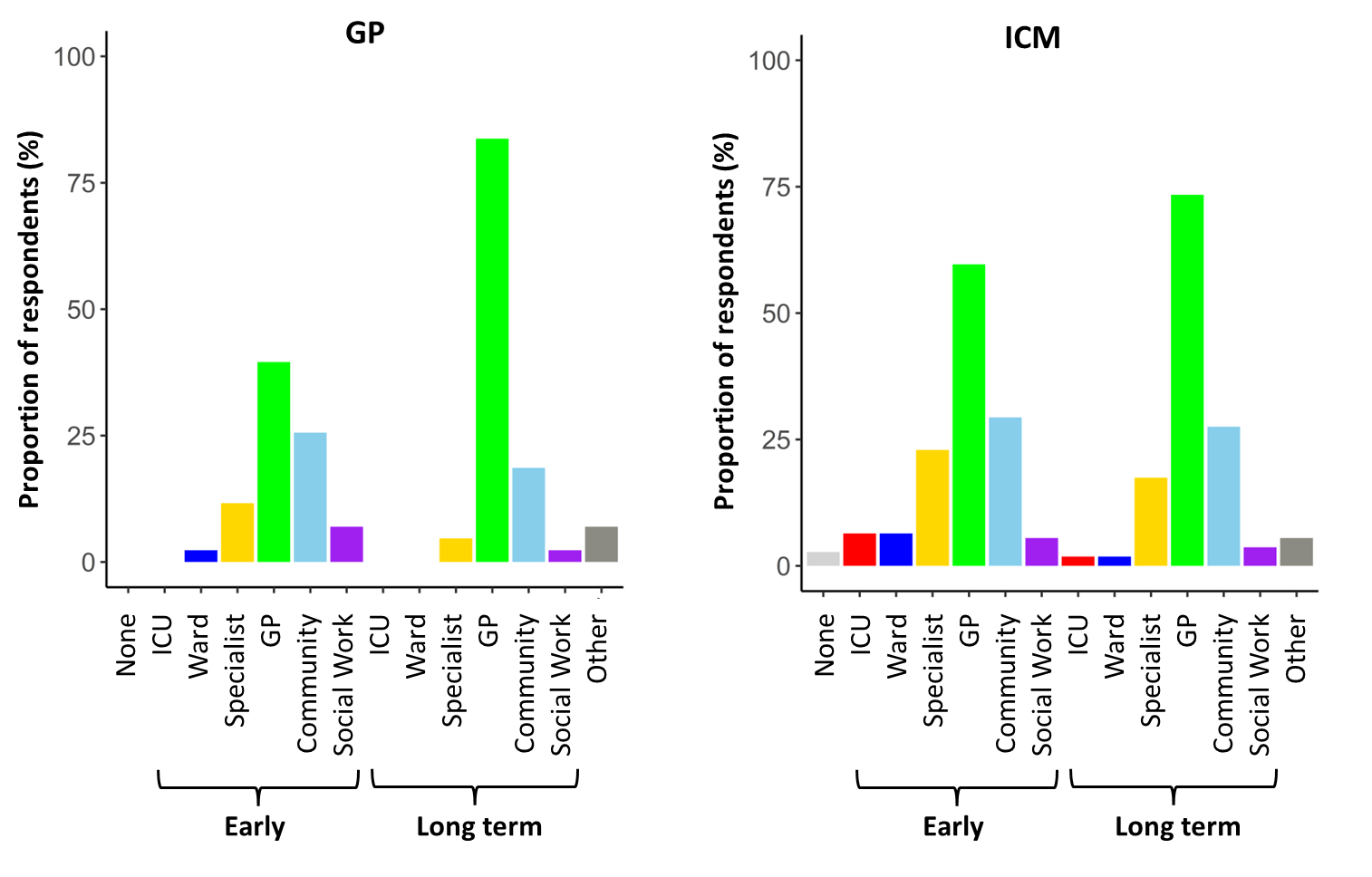
**

**Supplementary Figure 3b – Reponses to online questionnaire “Who do you think is responsible for follow-up of existing medical conditions (in this case COPD, hypertension and type 2 diabetes.**

**
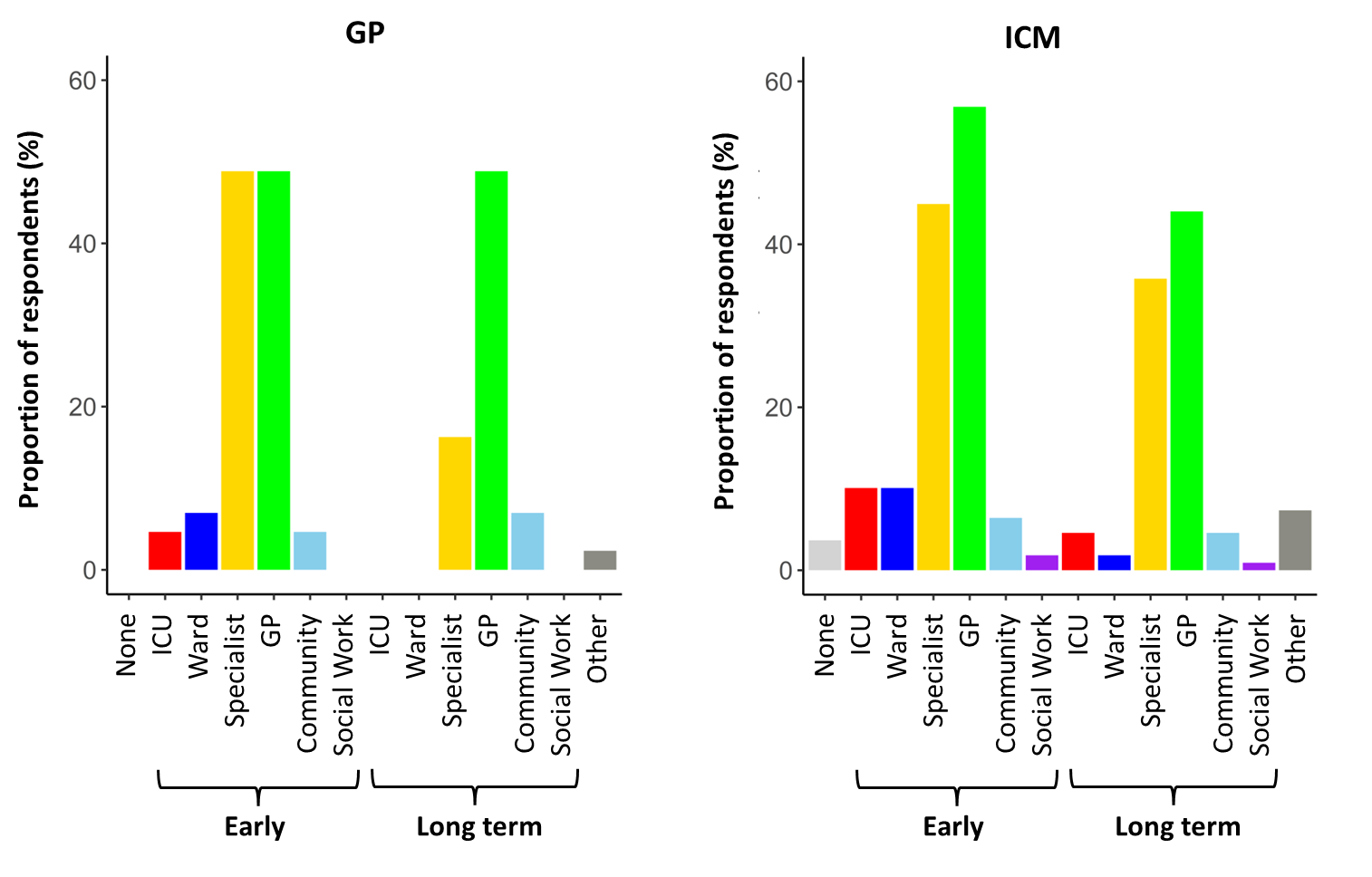
**

**Supplementary Figure 3c – Reponses to online questionnaire “Who do you think is responsible for follow-up of new renal impairment?”.**

**
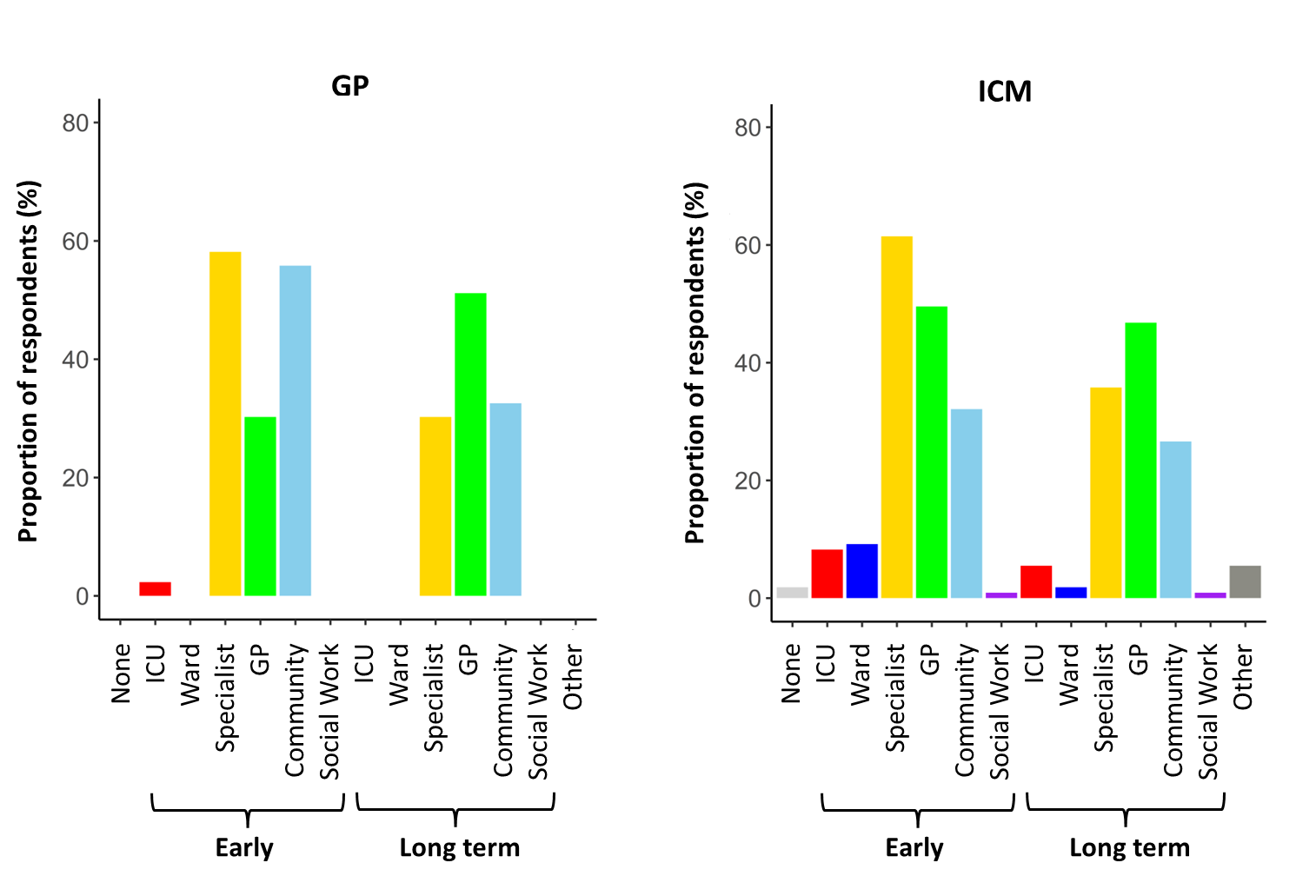
**

**Supplementary Figure 3d – Reponses to online questionnaire “Who do you think is responsible for follow-up of new heart failure?”.**

**
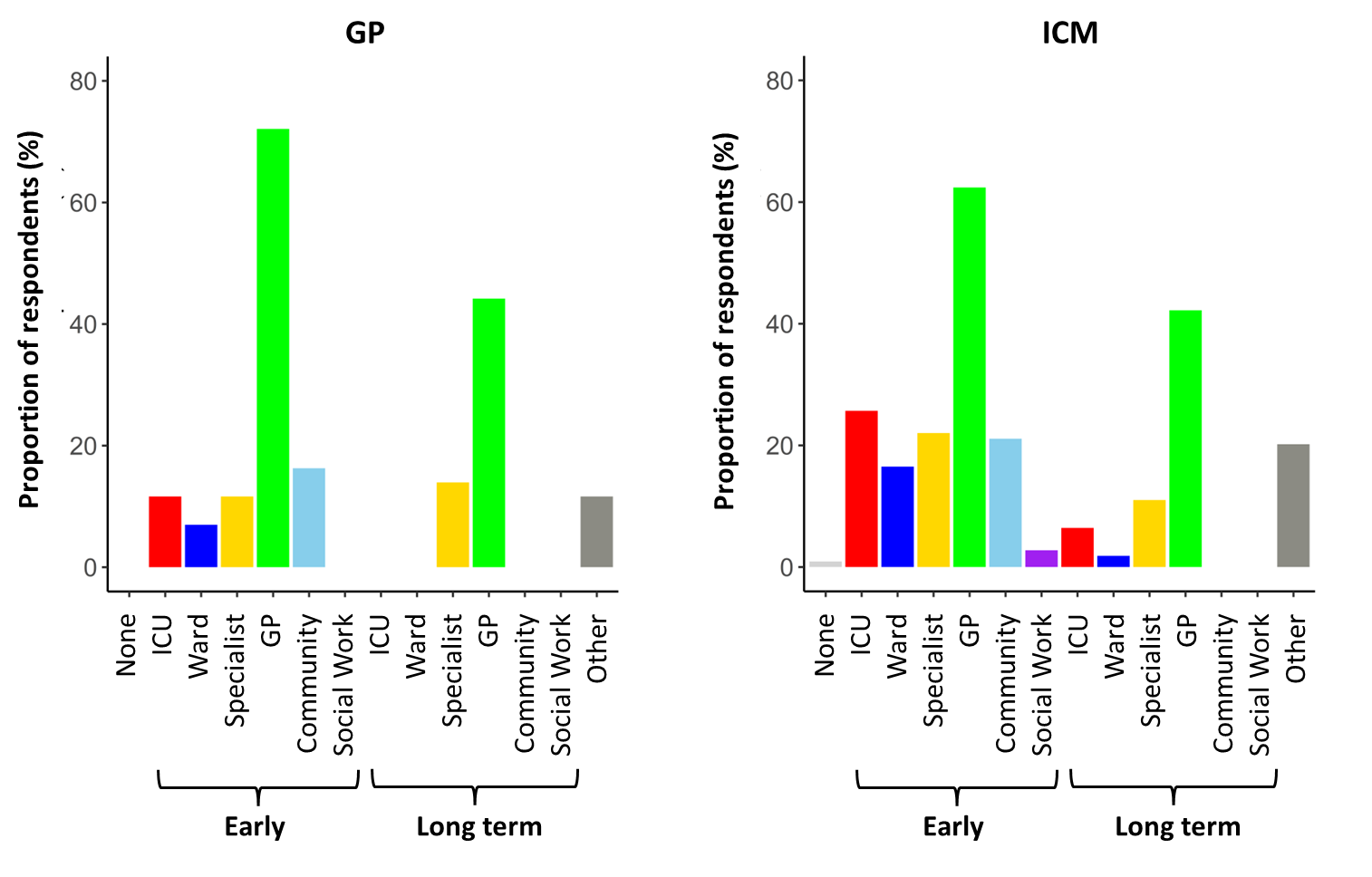
**

**Supplementary Figure 3e – Reponses to online questionnaire “Who do you think is responsible for follow-up of new medications?”.**

**
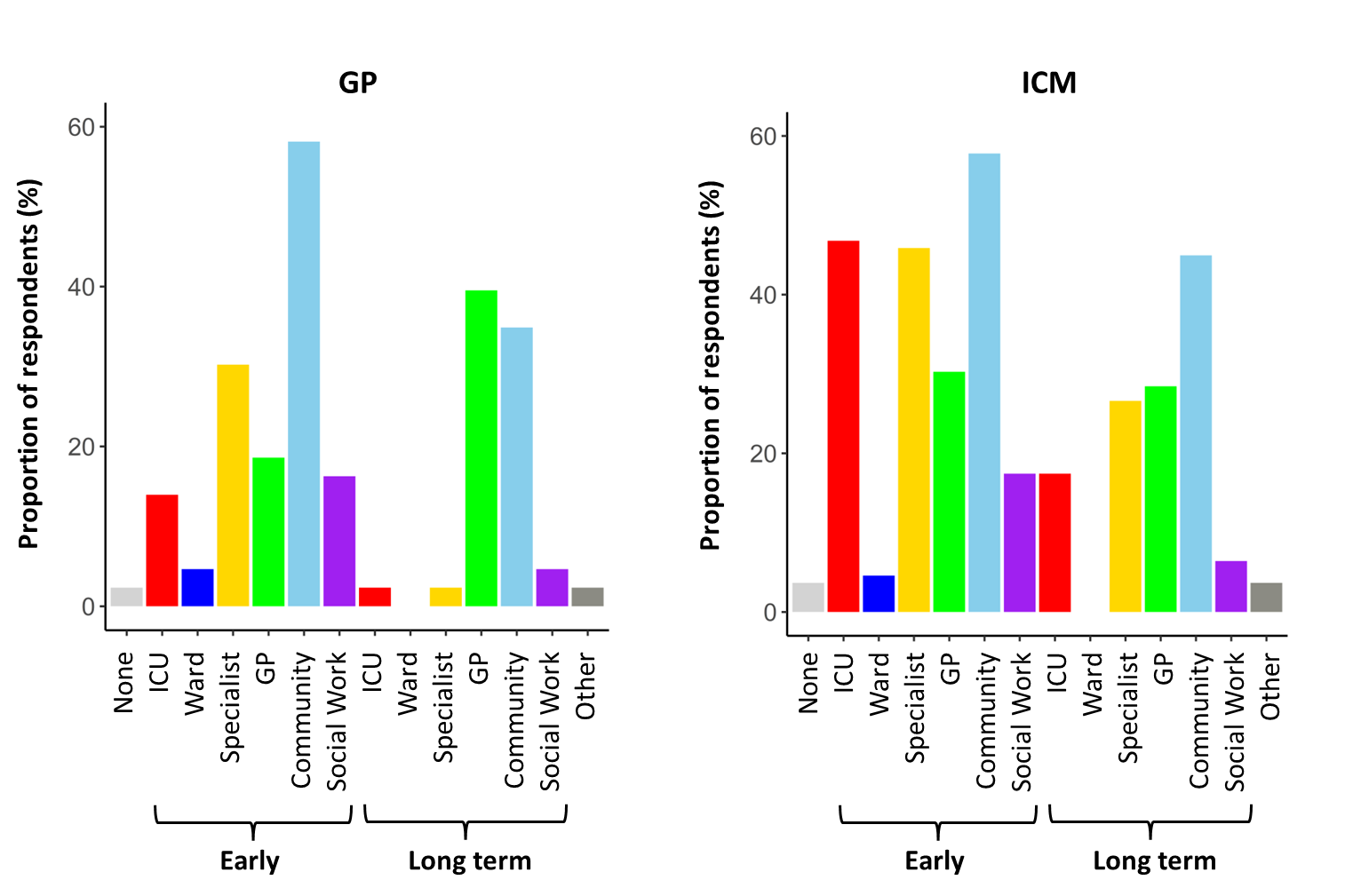
**

**Supplementary Figure 3f – Reponses to online questionnaire “Who do you think is responsible for follow-up of new physical weakness?”.**

**
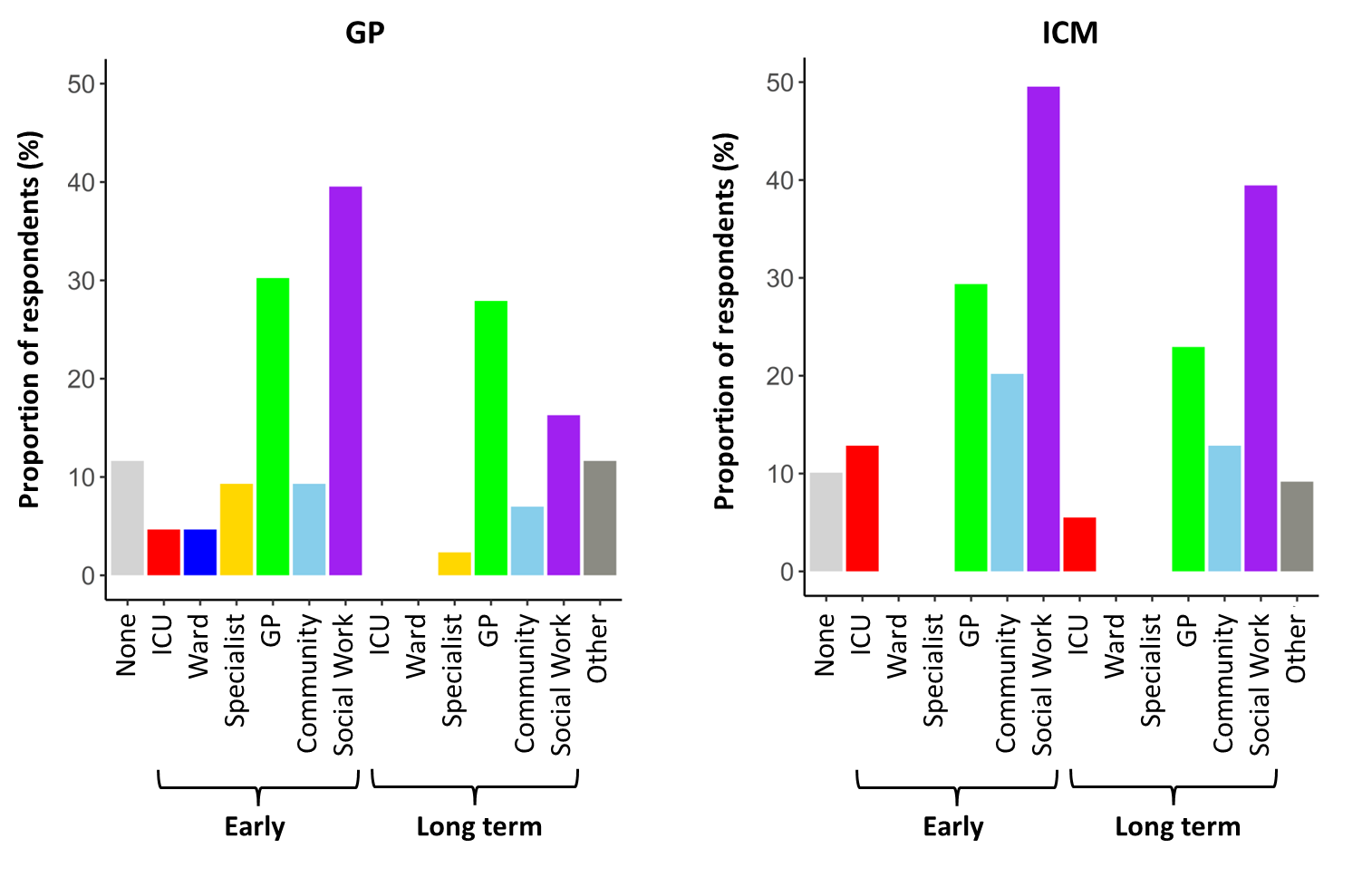
**

**Supplementary Figure 3g – Reponses to online questionnaire “Who do you think is responsible for follow-up of new employment disruption?”.**
