# Supplementary material for: Who is responsible for follow-up after critical illness? GP, ICU and patient perspectives
Source: Crit Care. 2025 Nov 14;29:489. doi: 10.1186/s13054-025-05724-w (PMC12619521; doi:10.1186/s13054-025-05724-w)
Supplement: Supplementary file 3 — Supplementary materials 3 [file 13054_2025_5724_MOESM3_ESM.docx]

**Consolidated criteria for reporting qualitative studies (COREQ): 32-item checklist**

| **No. Item** | **Guide questions/description** |  |
| --- | --- | --- |
| **Domain 1: Research team and reﬂexivity** |  |  |
| *Personal Characteristics* |  |  |
| 1. Inter viewer/facilitator | Which author/s conducted the interview or focus group? | JS. |
| 2. Credentials | What were the researcher’s credentials? E.g. PhD, MD | MRes. MPhil. |
| 3. Occupation | What was their occupation at the time of the study? | General Practitioner. Rehabilitation Medicine Specialty Registrar. PhD student. |
| 4. Gender | Was the researcher male or female? | Male |
| 5. Experience and training | What experience or training did the researcher have? | JS had experience with qualitative  methods including facilitating focus groups and  conducting interviews. |
| *Relationship with participants* |  |  |
| 6. Relationship established | Was a relationship established prior to study commencement? | The research team did not have any contact with participants prior to obtaining informed consent.  Researchers had no professional or ongoing  relationship with the participants |
| 7. Participant knowledge of the interviewer | What did the participants know about the researcher? e.g. personal goals, reasons for doing the research | Participants were aware the researcher (JS) is a General Practitioner and research fellow interested in developing interventions to improve outcomes for people following critical illness. |
| 8. Interviewer characteristics | What characteristics were reported about the inter viewer/facilitator? e.g. Bias, assumptions, reasons and interests in the research topic | Patients were aware of JS role as a General Practitioner and his research interest. |

| **Domain 2: study design** |  |  |
| --- | --- | --- |
| *Theoretical framework* |  |  |
| 9. Methodological orientation and Theory | What methodological orientation was stated to underpin the study? e.g. grounded theory, discourse analysis, ethnography, phenomenology, content analysis | Framework analysis informed by the domains of the Consolidated Framework for Implementation Research (CFIR) and Template for Intervention Description and Replication (TIDieR). |
| *Participant selection* |  |  |
| 10. Sampling | How were participants selected? e.g. purposive, convenience, consecutive, snowball | Purposive sample of staff based on their known expertise in supporting patient recovery after critical illness |
| 11. Method of approach | How were participants approached? e.g. face-to-face, telephone, mail, email | Email. |
| 12. Sample size | How many participants were in the study? | Interviews were completed with 9 ICM consultants and 5 GPs and 13 patients. Following initial analysis 2 workshops were completed with 3 and 5 patients respectively. |
| 13. Non-participation | How many people refused to participate or dropped out? Reasons? | NA |
| *Setting* |  |  |
| 14. Setting of data collection | Where was the data collected? e.g. home, clinic, workplace | Remote via Microsoft Teams. |
| 15. Presence of non-participants | Was anyone else present besides the participants and researchers? | No. However another member of the research team was available is required as per the distress protocol. |
| 16. Description of sample | What are the important characteristics of the sample? e.g. demographic data, date | Staff; Provision of care for patients recovering after critical illness.  Patients; Adults with lived experience of critical illness. |
| *Data collection* |  |  |
| 17. Interview guide | Were questions, prompts, guides provided by the authors? Was it pilot tested? | Topic guides which were pilot tested. |
| 18. Repeat interviews | Were repeat inter views carried out? If yes, how many? | No. |
| 19. Audio/visual recording | Did the research use audio or visual recording to collect the data? | Yes – audio and visual recording. |
| 20. Field notes | Were ﬁeld notes made during and/or after the inter view or focus group? | Yes. |
| 21. Duration | What was the duration of the inter views or focus group? | Interviews and focused groups lasted 30-60 minutes. |
| 22. Data saturation | Was data saturation discussed? | No. Information power. |
| 23. Transcripts returned | Were transcripts returned to participants for comment and/or correction? | No. |
| **Domain 3: analysis and ﬁndings** |  |  |
| *Data analysis* |  |  |
| 24. Number of data coders | How many data coders coded the data? | 2 (JS and RM) |
| 25. Description of the coding tree | Did authors provide a description of the coding tree? | No. |
| 26. Derivation of themes | Were themes identiﬁed in advance or derived from the data? | Both. |
| 27. Software | What software, if applicable, was used to manage the data? | NVIVO |
| 28. Participant checking | Did participants provide feedback on the ﬁndings? | No |
| *Reporting* |  |  |
| 29. Quotations presented | Were participant quotations presented to illustrate the themes/ﬁndings? Was each quotation identiﬁed? e.g. participant number | Yes.  Yes |
| 30. Data and ﬁndings consistent | Was there consistency between the data presented and the ﬁndings? | Yes. |
| 31. Clarity of major themes | Were major themes clearly presented in the ﬁndings? | Yes. |
| 32. Clarity of minor themes | Is there a description of diverse cases or discussion of minor themes? | Yes. |
